# Optical redox imaging of ANT1-deficient muscles

**DOI:** 10.1142/s1793545823500323

**Published:** 2024-01-30

**Authors:** He N. Xu, Ryan M. Morrow, Min Feng, Huaqing Zhao, Douglas Wallace, Lin Z. Li

**Affiliations:** * Britton Chance Laboratory of Redox Imaging, Department of Radiology, Perelman School of Medicine, University of Pennsylvania, Philadelphia, PA 19104, USA; † Center for Mitochondrial and Epigenomic Medicine, Children’s Hospital of Philadelphia Research Institute, Philadelphia, PA 19104, USA; ‡ Center for Biostatistics and Epidemiology, Lewis Katz School of Medicine at Temple University, Philadelphia, PA 19140, USA

**Keywords:** ANT1, redox ratio, flavoproteins

## Abstract

Adenine nucleotide translocator (ANT) is a mitochondrial protein involved in the exchange of ADP and ATP across the mitochondrial inner membrane. It plays a crucial role in cellular energy metabolism by facilitating the transport of ATP synthesized within the mitochondria to the cytoplasm. The isoform ANT1 predominately expresses in cardiac and skeletal muscles. Mutations or dysregulation in ANT1 have been implicated in various mitochondrial disorders and neuromuscular diseases. We aimed to examine whether ANT1 deletion may affect mitochondrial redox state in our established ANT1-deficient mice. Hearts and quadriceps resected from age-matched wild type (WT) and ANT1-deficient mice were snap-frozen in liquid nitrogen. The Chance redox scanner was utilized to perform 3D optical redox imaging. Each sample underwent scanning across 3–5 sections. Global averaging analysis showed no significant differences in the redox indices (NADH, flavin adenine dinucleotide containing-flavoproteins Fp, and the redox ratio Fp/(NADH+Fp) between WT and ANT1-deficient groups. However, quadriceps had higher Fp than hearts in both groups (*p* = 0.0004 and 0.01, respectively). Furthermore, the quadriceps were also more oxidized (a higher redox ratio) than hearts in WT group (*p* = 0.004). NADH levels were similar in all cases. Our data suggest that under non-stressful physical condition, the ANT1-deficient muscle cells were in the same mitochondrial state as WT ones and that the significant difference in the mitochondrial redox state between quadriceps and hearts found in WT might be diminished in ANT1-deficient ones. Redox imaging of muscles under physical stress can be conducted in future.

## 1. Introduction

Adenine nucleotide translocator (ANT) is a protein found in the mitochondria that facilitates the exchange of adenosine diphosphate (ADP) and adenosine triphosphate (ATP) across the inner membrane. This crucial function supports cellular energy metabolism by transporting cytoplasmic ADP into mitochondria and ATP synthesized within the mitochondria to the cytoplasm. ANT has four isoforms in human, ANT1, ANT2, ANT3, and ANT4,^1,2^ but only three in mice, i.e., ANT1, ANT2, and ANT4.^3–5^ ANT1 is primarily expressed in cardiac, skeletal muscles, and brain, with detectable low levels in other tissues. Dysregulation or mutations in ANT1 have been associated with a range of mitochondrial disorders and neuromuscular diseases, such as autosomal dominant progressive external ophthalmoplegia, mitochondrial myopathy, and cardiomyopathy as well as exercise intolerance.^6–8^

Previously, we created a global ANT1 “knockout” mice model to study the relevant mitochondrial diseases.^4^ This model showed ragged-red muscle fibers, a dramatic proliferation of mitochondria in skeletal muscle, and cardiac hypertrophy with mitochondrial proliferation. Mitochondria isolates from mutant skeletal muscle exhibited a severe defect in coupled respiration. However, the mitochondrial redox state of ANT1 mutant muscles has not been characterized.

The present optical redox imaging study aimed to examine at the tissue level whether ANT1 deficiency may significantly alter the mitochondrial redox state that regulates ATP generation. The redox pairs nicotinamide adenine dinucleotide (NAD^+^/NADH) and flavin adenine dinucleotide (FAD/FADH_2_) are essential co-enzymes in mitochondria that facilitate ATP generation thus regulating energy metabolism and mitochondrial reactive oxygen species (ROS) balance. FAD-containing flavoproteins (Fp) and protein-bound NADH are mainly found in mitochondria and are intrinsically fluorescent and emit green and blue photons, respectively, upon excitation at proper wavelengths; whereas NAD^+^ and FADH_2_ do not emit fluorescence. By imaging the intrinsic fluorescence of NADH and Fp, the optical redox imaging technique (ORI) can determine the levels of mitochondrial NADH and Fp and the mitochondrial redox state as represented by the redox ratio Fp/(NADH+Fp). The redox ratio also serves as an indicator of the cellular metabolic state and was found to correlate linearly with the intracellular redox ratio NAD^+^/(NADH+NAD^+^) *in vitro*.^9,10^ A lower value of Fp/(NADH+Fp) suggests a more reduced state, whereas a higher value suggests a more oxidized state. The Chance redox scanner uniquely allows the imaging of NADH and Fp fluorescence in tissues in 3D under liquid nitrogen temperature^11–13^ and has found wide applications to study tissue metabolic state under normal or pathological state.^14,15^ The present study employed the Chance redox scanner to examine snap-frozen heart tissues and quadriceps. Our study did not identify a significant change in the mitochondrial redox state associated with ANT1 deletion but did reveal that cardiac muscle cells were in a more reduced state than skeletal muscle cells in wild type (WT) mice but not in ANT1-deficient mice.

## 2. Materials and Methods

**Fig. 1.**
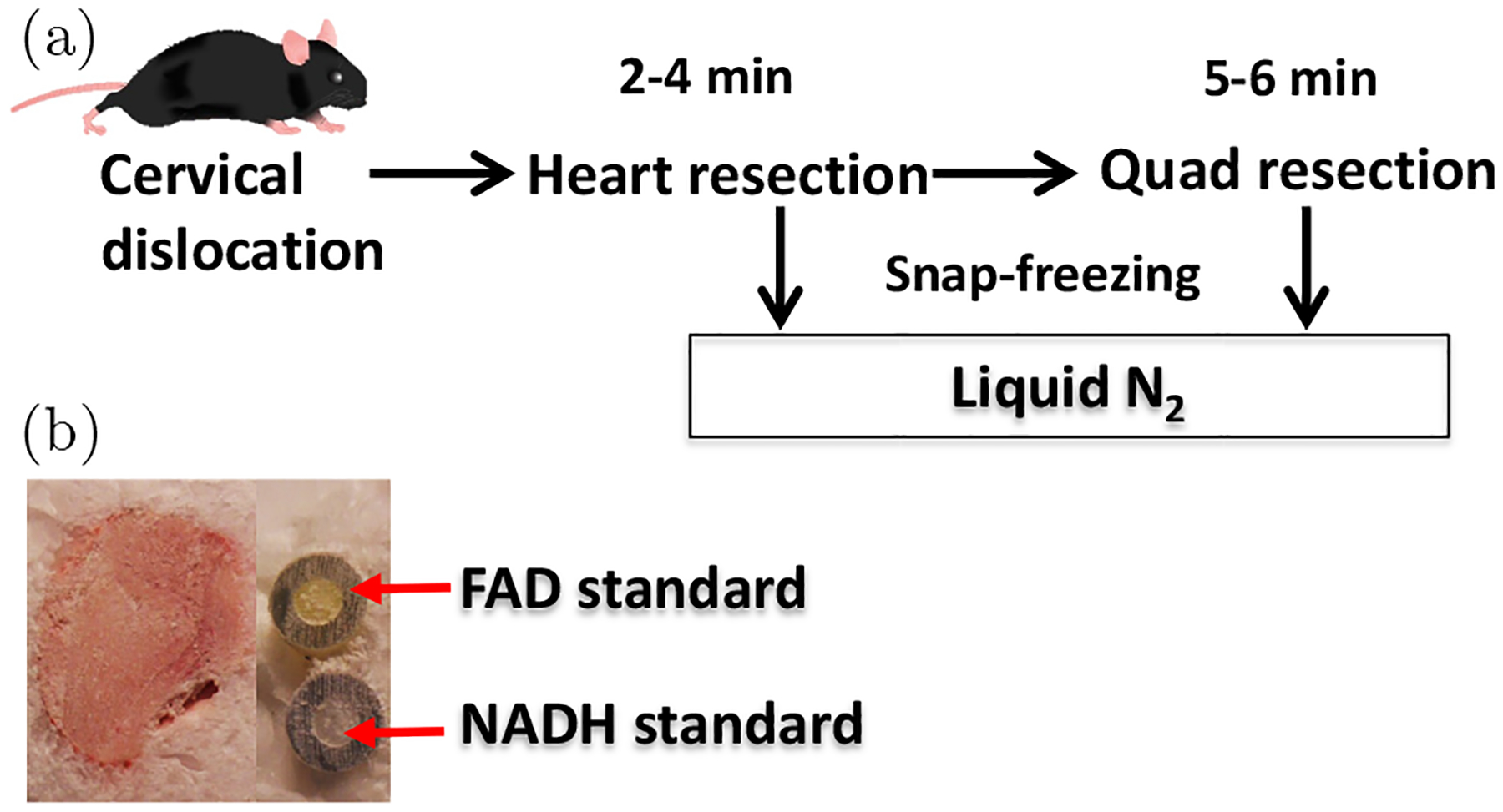
Tissue harvest, cryopreservation, and sample preparation for ORI. (a) Tissue harvest protocol; (b) Image of a tissue sample prepared with NADH and FAD reference standards placed next to it.

Age (3–4 months)-paired in-house bred mice of WT and ANT1 null on a C57BL background^4^ were provided by the Wallace lab at the Children’s Hospital of Philadelphia. The protocols and handling of the animals were approved by the Institutional Animal Care and Use Committee at both the Children’s Hospital of Philadelphia and the University of Pennsylvania. Within several hours after being received, the mice were sacrificed by cervical dislocation immediately followed by open-chest surgery for obtaining the heart and then the quadriceps. Immediately after removal from the body, the organs/tissues were snap-frozen using liquid nitrogen. The period between cervical dislocation and freezing of the excised heart and quadriceps ranged from 2–4 min to 5–6 min, respectively (Fig. 1(a)).

Sample preparation and redox scanning utilizing the Chance redox scanner have been described in our previous works.^15,16^ Briefly, the snap-frozen tissues were embedded in chilled mounting medium with the reference standards of FAD and NADH (100 *μ*M in PBS) embedded adjacently to the tissue sample (Fig. 1(b)) and stored in liquid nitrogen pending redox scanning. For redox scanning with the Chance redox scanner, the embedded tissue with adjacent reference standards were first milled flat and exposed for raster scanning. Each tissue sample was then scanned 3–5 sections spacing 200–400 *μ*m with an in-plane resolution of 200 *μ*m.

The raw redox imaging data were first processed by a customized computer algorithm using Matlab^®^ to obtain the images of nominal concentrations of Fp and NADH in reference to the embedded standards. The redox ratio images were generated pixel-by-pixel using the Fp and NADH concentration images. Global averaging was performed to obtain the mean value and the standard deviation (SD) of each redox index for each section. The mean values and the SDs were then averaged across all sections to obtain the values for the individual mouse and reported as one independent measurement. The difference in the redox indices (both mean values and SDs) between groups was compared via ordinary one-way ANOVA and the *p* values were adjusted by performing Bonferroni’s multiple comparisons test using Prism 9 (GraphPad). *P* < 0.05 was considered statistically significant.

## 3. Results

**Fig. 2.**
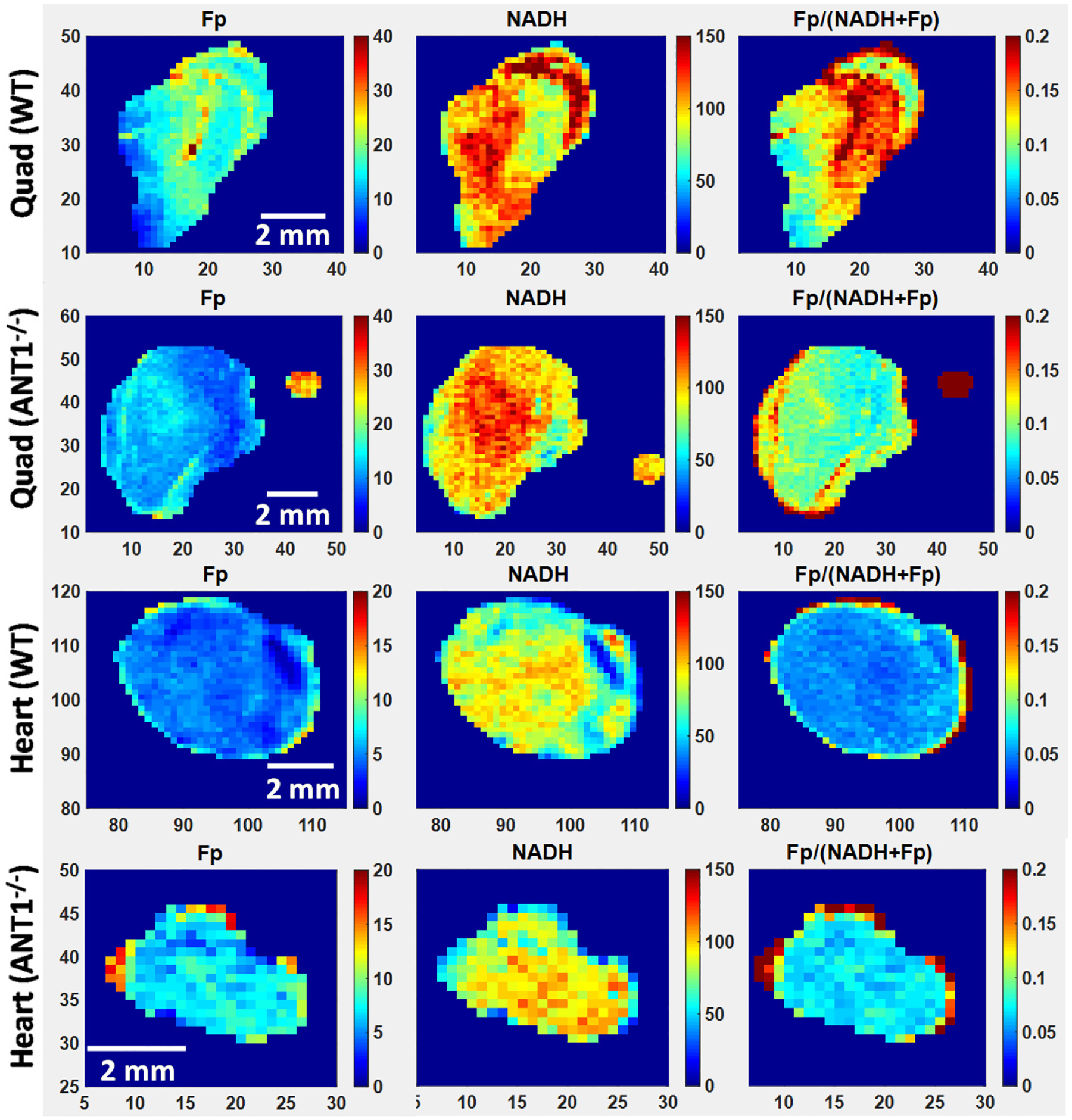
The representative redox images of quadriceps (Quad) and hearts from both WT and ANT1-deficient (ANT1^−/−^ mice. The color bars of Fp and NADH images indicate the nominal concentrations with red being more concentrated. The small round spots in the mutant quadricep images are the solution standards of either Fp or NADH. The color bars of the redox ratio images indicate the values of Fp/(NADH+Fp). The numbers along the *x* and *y* axes represent the spatial coordinates in pixels. All images have a spatial resolution of 200 *μ*m.

**Fig. 3.**
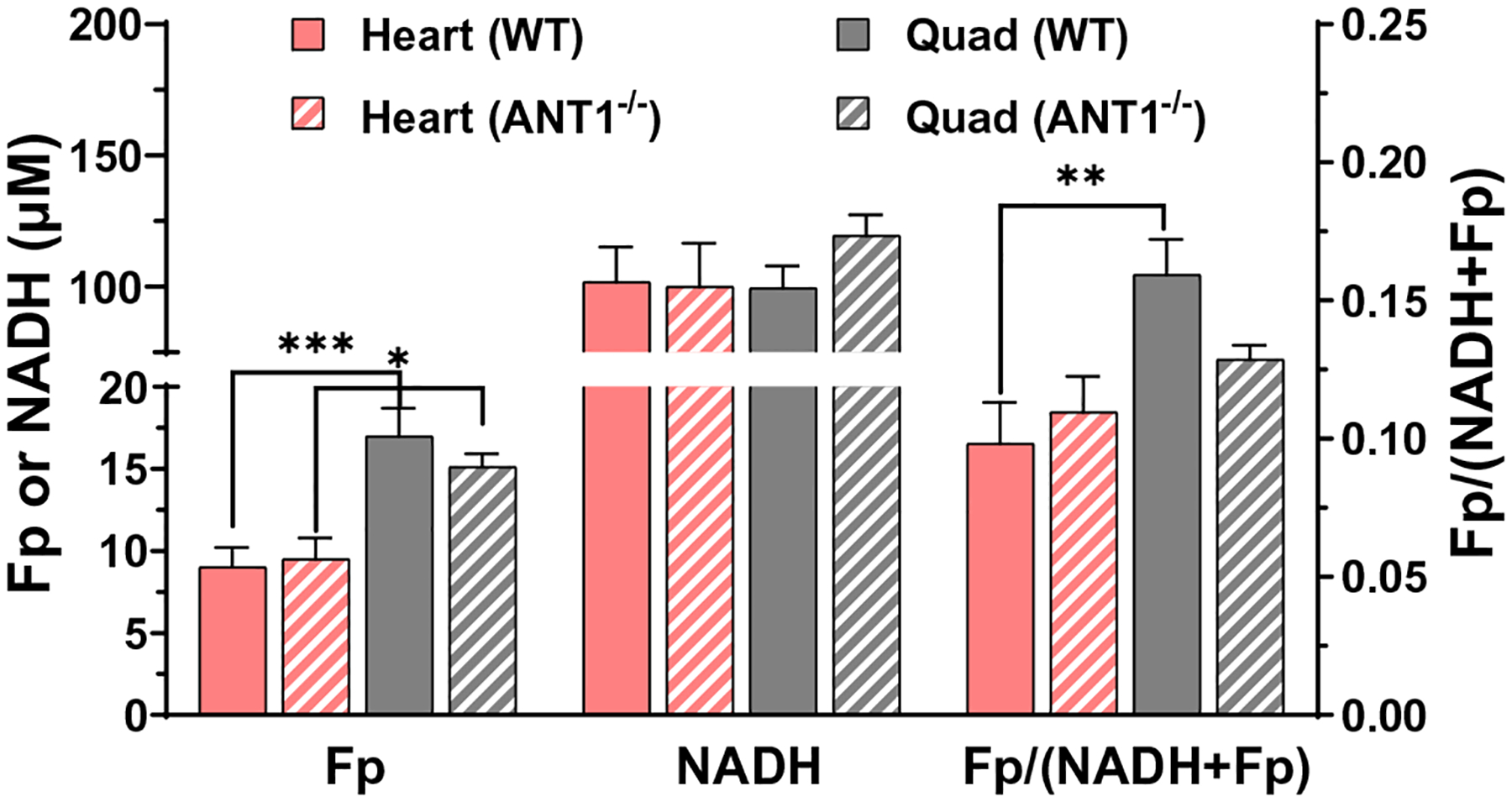
Quantitative analysis of the redox indices between genotypes and between heart and quadriceps within genotype. Mean ± SEM, where *n* = 8/genotype. *p < 0.05, **p < 0.01, ***p < 0.001.

The representative redox images are shown in Fig. 2. Imaging quantification shows that there was no significant change in either Fp, NADH, or the redox ratio due to ANT1 deletion (Fig. 3). However, comparing between different tissues of the same genotype, we found quadriceps had higher Fp levels than hearts in both genotypes with *p* = 0.0004 for WT and 0.01 for ANT1-deficient group. NADH, on the other hand, were similar in all cases for both genotypes. In WT mice, the redox ratio Fp/(NADH+Fp) was higher in quadriceps than in heart tissues (*p* = 0.004), indicating a more oxidized state in skeletal muscles. However, in mutant mice, the redox ratio difference between heart and quadriceps tissues was not significant (*p* = 0.8).

**Fig. 4.**
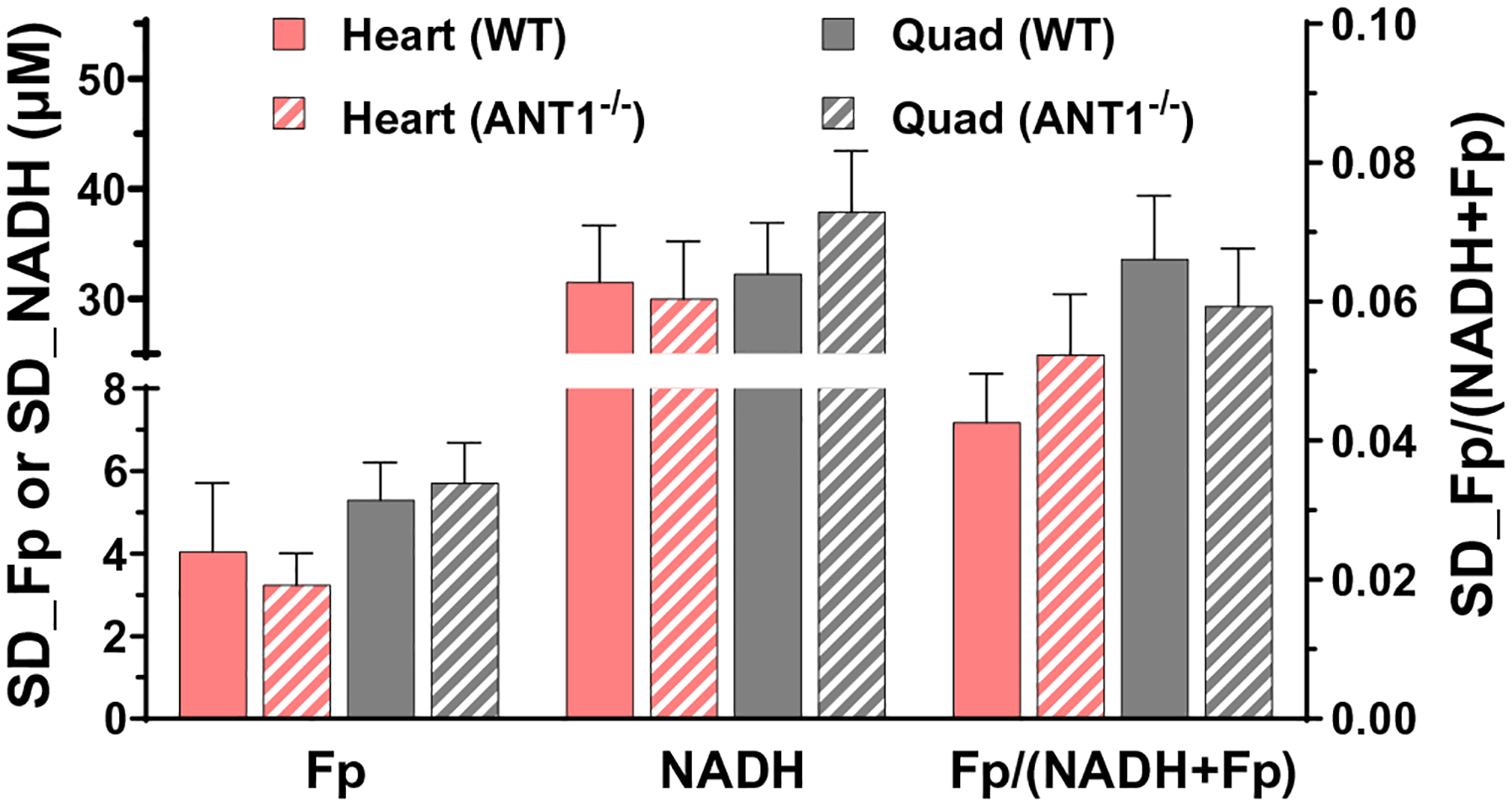
The redox heterogeneity indices as represented by the SDs in the *x* – *y* plane show no significant difference between genotypes or between heart and quadriceps within genotype, Mean ± SEM, where *n* = 8/genotype.

We also analyzed intra-tissue redox heterogeneity as represented by the SD of a specific redox index in the *x* – *y* plane. However, we did not identify a significant difference either between genotypes or between heart and quadriceps within genotype (Fig. 4).

## 4. Discussion

We previously showed that ANT1-deficient skeletal muscles had significant histological and ultrastructural differences compared to WT.^4^ Furthermore, ANT1-deficient mice exhibited severe exercise intolerance with striking difference in the respiratory exchange ratio (VCO_2_/VO_2_) whereas WT mice have constant VCO_2_/VO_2_ during exercise; ANT1-deficient mice have a declined VO_2_ resulting in an increasing VCO_2_/VO_2_ at increasing levels of work.^4^ We then found that the ANT1 knockout gastrocnemius had increased mitochondrial respiration compared to WT because the muscle was compensating by over proliferation of mitochondria. Yet, when oxygen consumption was normalized to mitochondrial citrate synthase activity the respiration defect in ANT1 mice was severe.^7^ Recently we discovered that exercise intolerance of ANT1-deficient mice was partially due to NAD^+^ limitation in skeletal muscle, which could be alleviated by NAD supplementation.^8^ In the study, NAD^+^ levels in the gastrocnemius muscle were quantified with NAD cyclin assay and reduced NAD^+^ level was revealed in ANT1-deficient muscle but the NAD^+^/NADH ratio was the same between WT and mutant muscle in either resting or exercise state. This is consistent with the finding in our present imaging study that no significant difference was observed in the redox ratio between WT and ANT1-deficient muscles.

Our imaging results reported here indicate that under normal non-stressful condition, the mutant muscle cells of both quadriceps and hearts can maintain the same mitochondrial redox state as WT muscle cells. Furthermore, for both genotypes, our analysis showed that the skeletal muscle had a significantly higher Fp level than the heart, suggesting a higher ROS level and/or more active mitochondrial metabolism in the skeletal muscle cells. Our analysis also showed a significantly larger Fp/(NADH+Fp) of quadriceps than that of the heart in WT but not in ANT1-deficient mice, suggesting the significant difference in the mitochondrial redox state between quadriceps and hearts found in WT might be diminished by ANT1 deletion. Redox imaging of muscles under physical stress can be carried out in future.

Intra-tissue heterogeneity can be quantified to reveal important biological/pathological information when the mean values quantified by global averaging fail to do so.^16–18^ For example, in one of our previous studies, we used the SDs of the redox indices to represent intra-pancreas redox heterogeneity and Gaussian curve fitting analysis of the redox ratio histograms of pancreatic tissues.^18^ Both heterogeneity analyses revealed that the PTEN null pancreases had significantly higher intra-tissue heterogeneity in the redox ratio than the control ones despite that their mean redox ratios were similar. In this study, as the tissue samples, especially the quadriceps showed apparent intra-tissue redox heterogeneity, we used the SD of a specific redox index in the *x* – *y* plane to represent the intra-tissue redox heterogeneity and performed statistical analysis, which yielded no significant difference either between or within genotypes.

## 5. Conclusions

Our optical redox imaging of the heart and skeletal muscle did not find a significant difference in the redox indices (NADH, Fp and the redox ratio Fp/(NADH+Fp)) between WT and ANT1-deficient mice under physical stress-free condition. In both genotypes, our analysis showed higher Fp in the skeletal muscle than that in the heart. Additionally, WT skeletal muscle was more oxidized than the heart, but this difference was diminished due to ANT1 deletion.
